# Clinical Characteristics Correlate With Outcomes of Immunotherapy in Advanced Non-Small Cell Lung Cancer

**DOI:** 10.7150/jca.49213

**Published:** 2020-10-18

**Authors:** Lan Huang, Li Li, Yingxu Zhou, Zhaoyang Yang, Meng Wang, Yina Gao, Yang Yang, Fang Yang, Bao Liu, Xuan Hong, Gongyan Chen

**Affiliations:** 1 Department of Medical Oncology, Heilongjiang Provincial Hospital, Harbin, China; 2 Department of Medical Oncology, Harbin Medical University Cancer Hospital, Harbin, China

**Keywords:** Non-small cell lung cancer, Immune checkpoint inhibitors, Immune-related adverse events (irAEs), Peripheral blood biomarker, clinical outcome

## Abstract

Considering the existing indicators are not sufficient to predict the patient's response to immune checkpoint inhibitors (ICIs), we conducted this study to evaluate the efficacy and safety of ICIs in advanced non-small cell lung cancer (NSCLC) patients, and to determine prognostic factors of ICIs. In this study, 61 patients diagnosed with advanced NSCLC who underwent ICIs were recruited. The univariate analysis revealed the number of metastatic sites, immune-related adverse events (irAEs) (≥ G2) and best response were significantly associated with both progression-free survival (PFS) and overall survival (OS). Peripheral blood biomarkers, including post-treatment neutrophil-to-lymphocyte ratio (NLR) and CEA levels were also associated with PFS, but not OS. The irAEs (≥ G2), best response and age were confirmed as independent predictors of a prolonged survival by multivariate analysis. The development of irAEs ≥ G2 correlated with a survival benefit in patients with advanced NSCLC (median PFS: 7.1 months vs. 4.6 months,* P* = 0.013). Thus, we concluded that identifying predictors of benefit from ICIs treatment will help to further extend patient survival in advanced NSCLC.

## Introduction

Non-small cell lung cancer (NSCLC) is a major cause of cancer-related deaths worldwide 1. In addition, most patients have been identified as advanced or metastatic NSCLC at the time of initial diagnosis. Platinum-based chemotherapy has been the preferred option for patients with NSCLC since 1990s. Patients harboring genetic mutations (i.e. EGFR, ALK, ROS-1) can benefit from the appropriate targeted therapies 2. With the rapid development of cancer immunotherapy, accumulating evidences indicate that it has become the fourth strategy of cancer treatment.

Immune checkpoint inhibitors (ICIs), particularly those acting on the anti-PD-1 (programmed cell death-1) / PD-L1 (programmed cell death-Ligand 1) and anti-CTLA-4 (cytotoxic T-lymphocyte antigen-4) axis, have greatly improved survival in patients with advanced NSCLC. To date, this unprecedented clinical benefit has driven the development of immunotherapy to first-line treatment 3. In clinical trials of CheckMate and Keynote series, the efficacy of anti-PD-1 antibodies as second-line therapy was better than standard chemotherapy in NSCLC 4-6. In addition, data from OAK and PACIFIC studies showed that patients with NSCLC benefit from anti-PD-L1 antibodies 7, 8. As a result, anti-PD-1 and anti-PD-L1 antibodies have been approved for second- or later-line and maintenance therapy after chemoradiotherapy.

According to reports, several baseline tumor features, including PD-L1 expression, tumor mutational burden (TMB) and CD8^+^ T cells infiltration have been shown to be associated with response to ICIs. However, emerging evidence shows that patients with low or negative PD-L1 expression may also have a good response to ICIs treatment 9,10. Considering the existing indicators are not sufficient to predict the patient's response to ICIs. There is an urgent need to explore the selection criteria for advanced NSCLC patients who could benefit from ICIs therapy.

Moreover, due to the application of ICIs, physicians are required to manage a series of new side effects, the so-called immune-related adverse events (irAEs). The irAEs result from an aberrant activation of T-cell and B-cell mediated pathway, elicited by ICIs, leading to autoimmunity disorders 11, 12. According to reports, nearly 70% of patients receiving anti-PD-1/PD-L1 antibodies will develop irAEs of any grade 13. For patients receiving anti-CTLA-4 antibody, the occurrence of irAEs is up to 90% 14. Thus, we retrospected and analyzed the patient's irAEs in this study. Overall, we aimed to investigate the factors that predict the clinical outcome of ICIs treatment and to describe relevant irAEs in advanced NSCLC.

## Materials and Methods

### Patients

This retrospective cohort study was conducted at the Harbin Medical University Cancer Hospital (Harbin, China). Patients who received immunotherapy between August 2016 and December 2018 were enrolled. The observational period for evaluation of efficacy and safety was from treatment initiation to disease progression. In this study, nivolumab and pembrolizumab were administered as anti-PD-1 antibody treatments, atezolizumab as anti-PD-L1 antibody treatment, ipilimumab as anti-CTLA4 antibody treatment. Some patients received the pre-treatment with standard chemotherapy based on platinum and paclitaxel/pemetrexed. The patients enrolled in this study fulfilled the following characteristics:(I) pathologically confirmed advanced NSCLC; (II) clinical stage was IIIB-IV; (III) ECOG score was 0-1 (exclude one person is 2); (IV) received at least 1 cycle of ICI agents, regardless of pretreatment line; (V) EGFR, ALK and ROS genes are wild-type (exclude 8 patients).

The following data were collected: clinical and treatment characteristics, local and distant metastasis sites, laboratory data before immunotherapy, progression-free survival (PFS) time, overall survival (OS) time, the response to therapy, and irAEs. Performance status (PS) was assessed using the Eastern Cooperative Oncology Group (ECOG) scores criteria. Metastases in more than two organs including pleural, contralateral lung or distant organs was defined as ≥ 3 metastasis sites. The definition of NLR is according to previous reports, and the NLR cut-off value was set as 5 15, 16. The response to therapy (complete response (CR), partial response (PR), stable disease (SD), progressive disease (PD)) was assessed according to the Response Evaluation Criteria in Solid Tumors (RECIST) (version 1.1). The irAEs were evaluated according to the National Cancer Institute Common Toxicity Criteria for Adverse Events (version 4.0). This study was approved by the Scientific and Ethical Committee of the Harbin Medical University Cancer Hospital. All patients provided written informed consent.

### Statistical analysis

Descriptive statistics were used to analyze and report clinical variables. PFS was measured as the time between initiation of ICIs treatment to the first time disease progression, or death for any cause. OS was calculated as the time from the beginning of ICIs treatment to death for any cause or the last follow-up visit with no evidence of progression. PFS and OS were estimated using the Kaplan-Meier method and analyzed using the log-rank test.

A univariate analysis was performed according to sex (male vs. female), age (< 65 years vs. ≥ 65 years), smoking history (smoker vs. non-smoker), tumor histology (squamous cell carcinoma vs. adenocarcinoma), ICIs treatment (anti-PD-1 vs. anti-PD-L1 vs. anti-CTLA4 combined with anti-PD-1), clinical stage (IIIB vs. IV), baseline ECOG score (0-1 vs. 2), number of metastatic sites at the beginning of immunotherapy (< 3 vs. ≥ 3), irAE class I (any irAE vs. no irAE), irAE class II (irAE ≥ G2 vs. irAE < G2 or no irAE) and the response to therapy (CR+PR vs. SD vs. PD). Cox proportional hazard model is applied to univariate test, where significant variables are used for multivariate analysis. Hazard ratios (HR) were presented with 95% confidence intervals (CI). Pearson's chi-squared test was performed in the analysis of patient and treatment characteristics associated with the response to and PFS of ICIs treatment; and Fisher's exact test was used when needed. All analyses were two-sided and values of *P* < 0.05 were considered statistically significant. Statistical analyses were performed using SPSS (version 17.0, USA) software package.

## Results

### Patient characteristics

A total of 61 patients with advanced NSCLC were enrolled. The population included 38 male and 23 female. Median age was 57 years (range: 20-75 years), and 27 patients (44.3%) had a history of smoking. Primary tumors were squamous cell carcinoma in 16 cases (26.2%), adenocarcinoma in 45 cases (73.8%). ECOG score at the initial of immunotherapy was 0-1 score in 60 patients (98.4%). There are 10 patients with ≥ 3 metastatic sites, and 51 patients with < 3 sites. Ten patients received immunotherapy as first-line treatment, with 5 receiving nivolumab combined with chemotherapy (based on platinum and paclitaxel), with 5 receiving pembrolizumab, with 3 receiving atezolizumab, with 4 receiving nivolumab combined with ipilimumab. The remaining patients received single-agent ICIs as second-line (15 cases), third-line (10 cases), fifth-line (2 cases), respectively. Five of 61 patients received cytotoxic chemotherapy combined with immunotherapy. For pretreatment strategy, paclitaxel and platinum are most frequently administered in patients with squamous cell carcinoma (7/8; 87.5%), and pemetrexed and platinum are commonly used in patients with adenocarcinoma (32/36; 88.9%). Twenty-four patients (39.3%) experienced irAEs. Patient characteristics are detailed in Table 1.

### Univariate and multivariate analysis for the PFS and OS

Univariate and multivariate analysis for the PFS and OS were detailed in Table 2 and Table 3, respectively. The results of univariate analysis suggested that metastasis sites (< 3) (*P* = 0.039; *P* = 0.012), irAE (≥ 2 grade, short for G2) (*P* = 0.395; *P* = 0.025) and response to therapy (*P* < 0.001;* P* = 0.009) were correlated with significant prolonged PFS and OS, respectively. In addition, age (≥ 65 yr) (HR = 3.878, 95% CI 1.686-8.921, *P* = 0.001) was significantly correlated with inferior OS. The elevated pretreatment CEA level (≥ 5 ng/ml) (HR = 0.437, 95% CI 0.225-0.846, *P* = 0.014) was associated with significant shorter PFS. Although no correlation between pre-treatment neutrophil-to-lymphocyte ratio (NLR C1) values and PFS was found in patients receiving ICIs (cut-off value = 5; *P* = 0.431, HR = 0.709, 95% CI: 0.301-1.670), while the higher NLR of the fourth cycle of treatment was significantly associated with inferior PFS (*P* = 0.002, HR = 3.060, 95% CI: 1.521-6.156) (Table 2). In the further multivariate analysis, irAE (≥ G2) (HR = 0.240, 95% CI 0.082-0.704, P = 0.009) and response to therapy (HR = 10.435, 95% CI 3.677-29.613, *P* < 0.001) were associated with prolonged PFS, and age < 65 yr (HR = 5.45, 95% CI 1.982-14.98, P = 0.001) was related to superior OS. The above results suggested these characteristics might serve as predictive indicators for advanced NSCLC patients treated with ICIs (Table 3).

**Figure 1:**
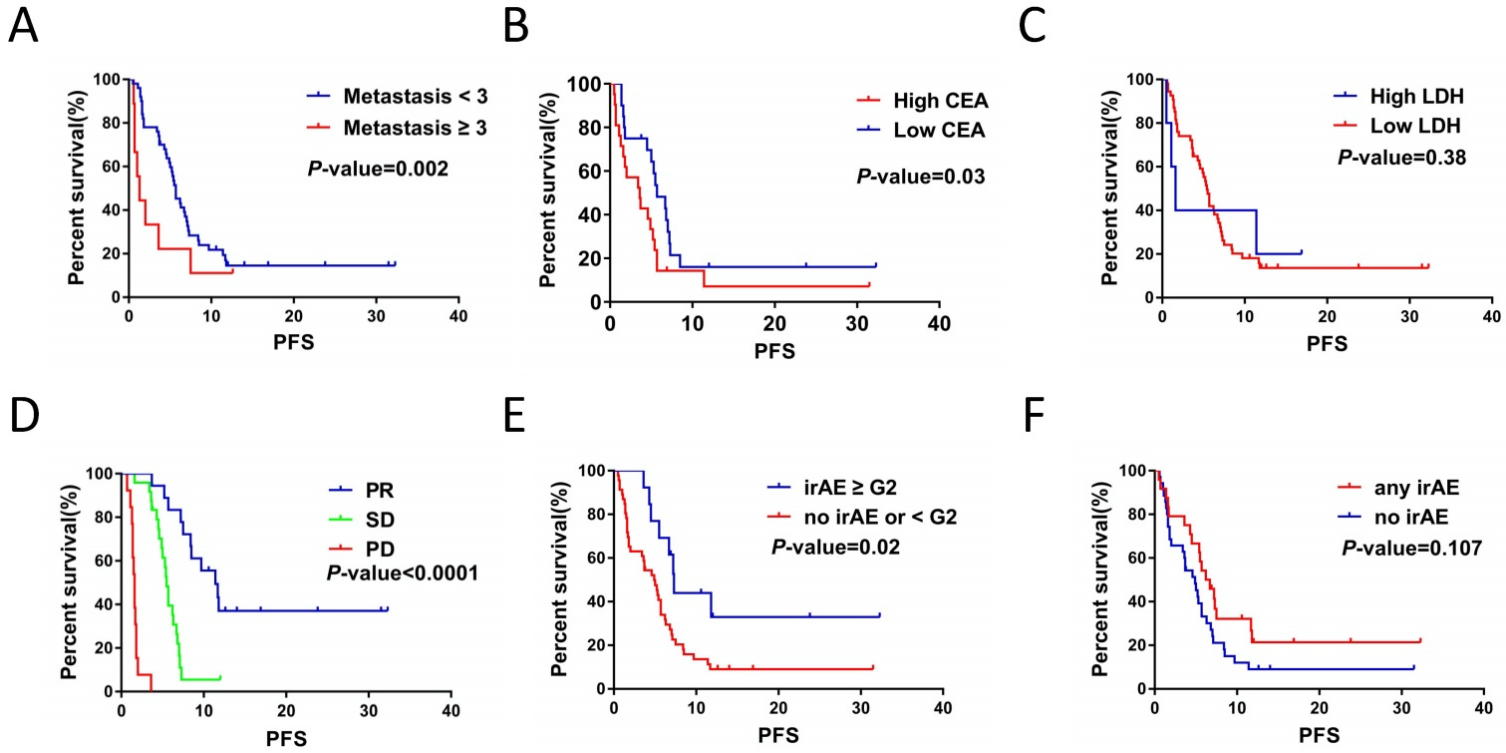
The correlations between clinicopathological characteristics, hematological markers, clinica response and PFS of the patients with ICIs treatment. A. Numbers of metastasis site affected the PFS for ICIs treatment (*P* = 0.002). B. CEA levels affected the PFS for ICIs treatment (*P* = 0.03). C. Serum LDH levels had no significant impacts on PFS for ICIs treatment (*P* = 0.38). D. Response to ICIs affected the PFS for ICIs treatment (*P* < 0.001). E. irAE (< G2 / ≥ G2) was associated with PFS for ICIs treatment (*P* = 0.02). F. irAE (no/any irAE) had no significant impacts on PFS for ICIs treatment (*P* = 0.107).

**Figure 2:**
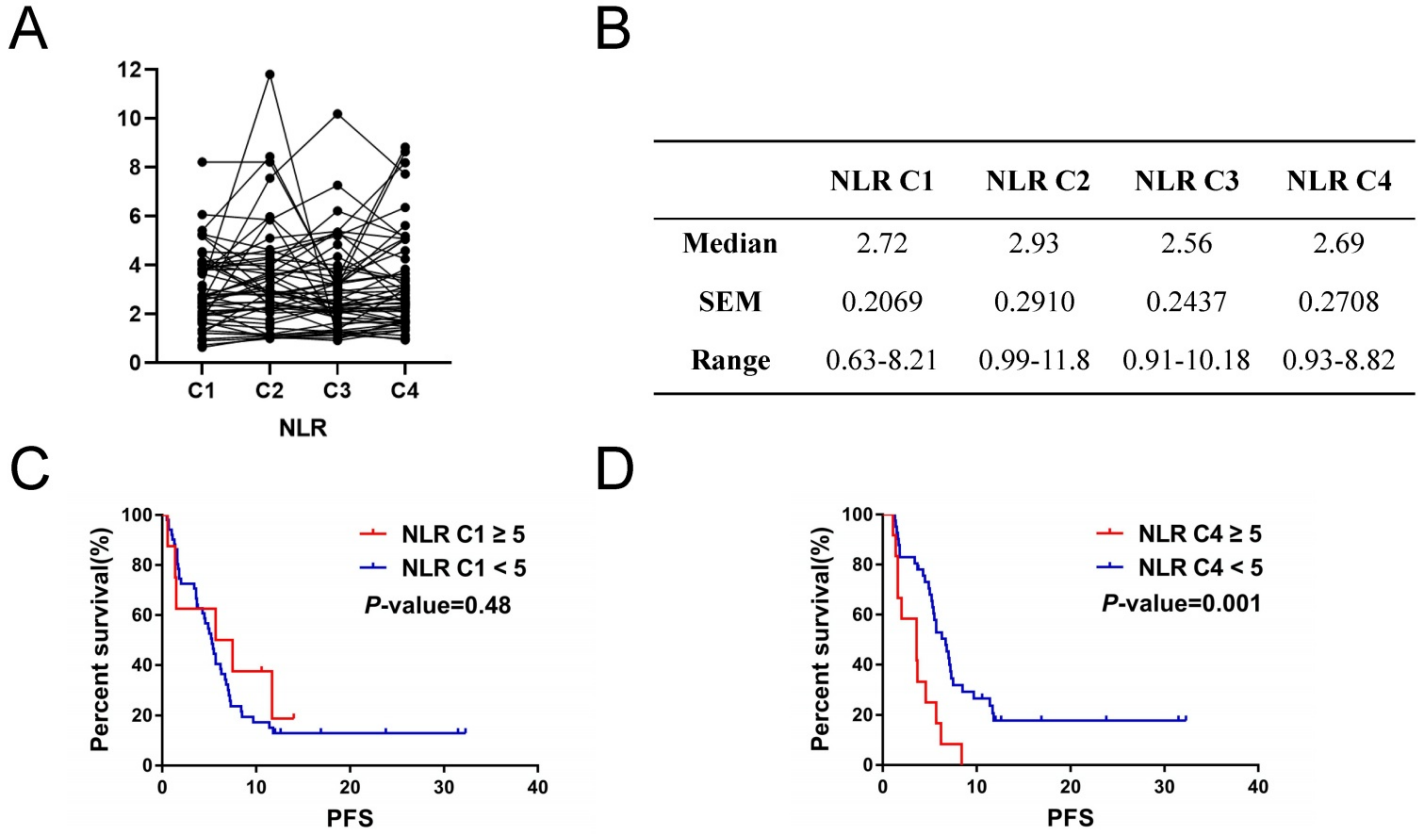
Inflammation-related factor NLR had prognostic value for PFS of NSCLC patients with ICIs treatment. A. and B. Variation of NLR volumes from C1 to C4. C. Cycle 1 neutrophil-to-lymphocyte ratio (NLR C1) had no significant impacts on PFS of ICI treatment (*P* = 0.48). D. NLR C4 correlated with PFS of ICI treatment (*P* = 0.001).

Kaplan-Meier survival analysis showed that metastasis sites ≥ 3 (*P* = 0.002), high CEA (*P =* 0.03) and response to therapy (*P <* 0.001) were closely related to poor PFS (Figure 1A, 1B and 1D). For serum lactate dehydrogenase (LDH), we set the cut-off value at 1.5 times of upper normal limit (UNL). However, we did not find its correlation with PFS (Figure 1C). We further analyzed the correlation between irAEs and prognosis of ICIs treatment and the irAEs class II (classified as < or ≥ G2), but not irAEs class I (classified as no/any irAEs), was significantly correlated with the PFS (Figure 2E and 2F). In this study, we also focused on the prognostic value of NLR. Figure 2A and 2B showed the variation of NLR from C1 to C4. No significantly differences of median NLR were found (*P =* 0.608). As shown in Figure 2C and 2D, the NLR in the fourth cycle (NLR C4), not the pre-treatment NLR (NLR before the first cycle of treatment; NLR C1 for short; *P* = 0.48), was significantly associated with PFS of ICIs treatment (*P =* 0.001).

**Figure 3:**
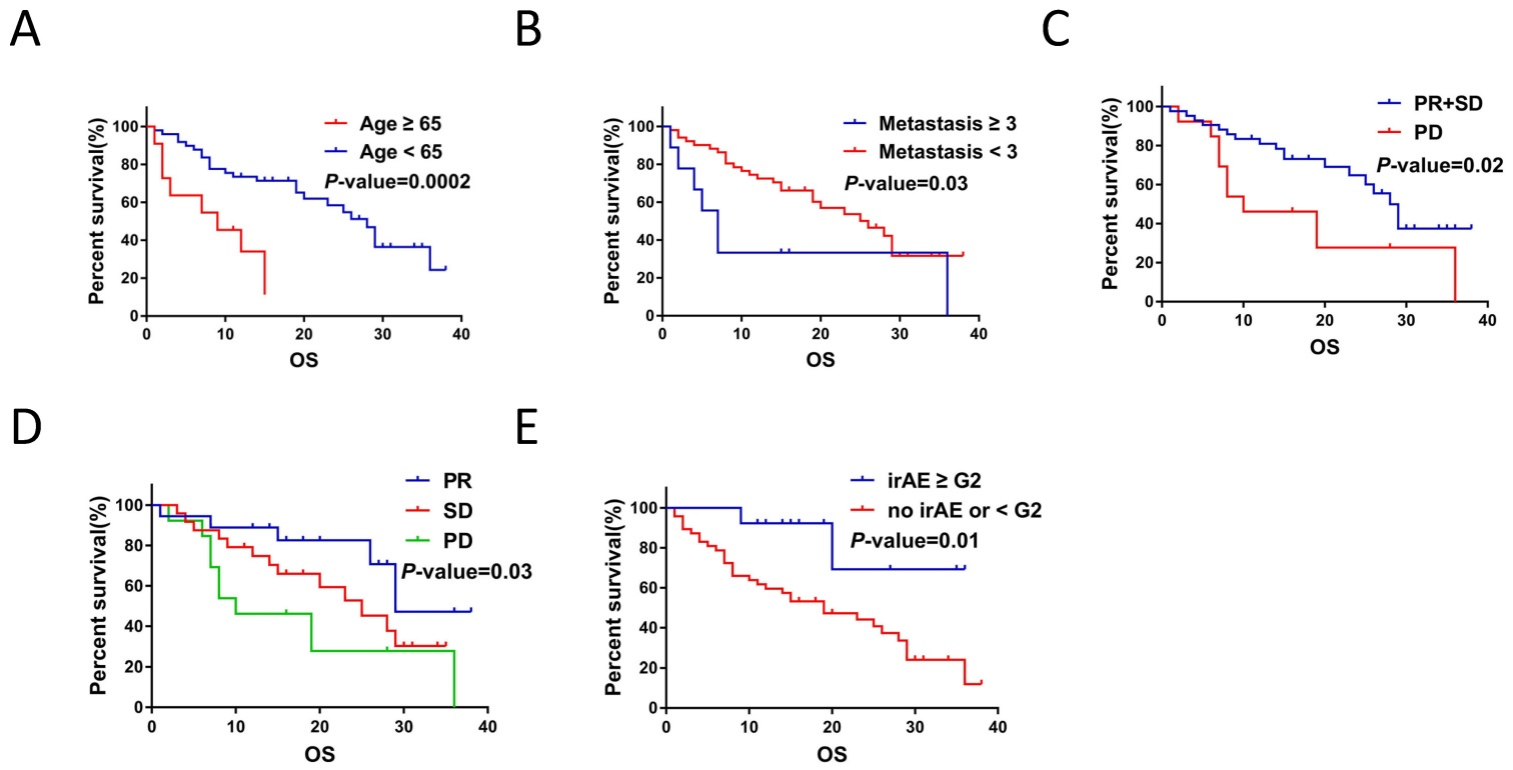
Clinicopathological and treatment characteristics were associated with OS of NSCLC patients with ICIs treatment. A. Age correlated with the OS for ICIs treatment (*P* < 0.001). B. Number of metastatic organ affected the OS of ICIs treatment (*P* = 0.03). C. Treatment response to ICIs affected OS of NSCLC patients (*P* < 0.001). D. Response to ICIs affected the OS of ICIs treatment (*P* = 0.03). E. irAE (< G2 / ≥ G2) affected the OS of ICIs treatment (*P* = 0.01).

Furthermore, Kaplan-Meier survival analysis showed that age < 65yr (*P <* 0.001), metastasis sites < 3 (*P* = 0.03), treatment response (*P* = 0.02), best response (*P* = 0.03) and irAEs (≥ G2) (*P* = 0.01) were associated with prolonged OS (Figure 3A-3E).

### Clinical outcome of immunotherapy

The median PFS and OS of all patients were 5.2 months (1.7 months-32.3 months) and 15 months (0 months-38 months), respectively. The median time to achieve optimal response is 1.8 months (1.1 months-29.3 months). Among 61 patients, no CR was achieved, in addition, 18 cases (29.5%) achieved PR, 24 cases (39.3%) achieved SD and 13 cases (21.3%) achieved PD.

**Figure 4:**
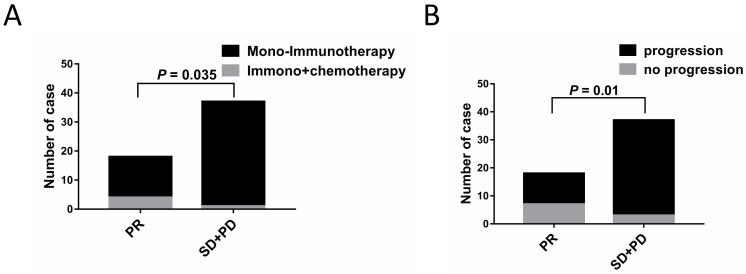
Patient and treatment characteristics were associated with ICIs treatment response. A. Differences in treatment strategies between ICIs treatment responses (*P* = 0.035). B. Differences in progression situation between ICIs treatment responses (*P* = 0.01).

The correlations between the clinical outcomes and characteristics are shown in Table 4. No significant differences between the PR vs. SD+PD groups were observed in terms of age, sex, smoking history, histology, clinical stage, metastasis sites, irAEs, CEA, NLR C4, PLT and LDH levels. As shown in Table 4, there seemed to be more patients receiving immunotherapy combined chemotherapy and low rates of progression in PR group (Figure 4).

### Profiles of immune-related adverse events

Different from standard chemotherapy and targeted therapy, ICIs treatment develops unique therapeutic side effects called immune-related adverse events. Overall, 24 patients (39.3%) developed irAEs at any grade and 12 (19.7%) patients had ≥ G2 events (Table 1). As shown in Table 5, the irAEs were categorized on the basis of the organ/system involving skin, endocrine, respiratory system, hepatic system, cardiac system (including premature ventricular beats and myocardial ischemia), gastrointestinal and others irAEs (including fever, fatigue, anemia, thrombocytopenia, albumin reduction, dizziness, hyponatremia, hypochloremia, creatinine increase, uric acid increase, xerophthalmia, pigmentation). In addition, we analyzed irAEs based on different immunotherapeutic drugs. As observed in Table 5, all 4 patients who received combination therapy with anti-PD-1 and anti-CTLA4 developed varying degrees of irAEs, significantly higher than patients who received single therapy (anti-PD-1, 50%; anti-PD-L1, 18.5%). The details were summarized in Table 5.

As mentioned above, the overall median PFS was 5.2 months. Median PFS of patients who experienced irAEs ≥ G2 was 7.1 months (range from 3.6 months to 32.3 months), whereas median PFS of patients who experienced no irAEs or irAEs < G2 was 4.6 months (range from 0 months to 31.5 months) (*P* = 0.013, HR= 0.264, 95% CI 0.092-0.751). Therefore, the occurrence of irAEs ≥ G2 may indicate a better clinical outcome (Figure 2D).

Moreover, our analysis showed that anti-PD-1 therapy has a higher incidence of any irAE (*P* = 0.013) than anti-PD-L1therapy, while no difference in occurrence of irAEs ≥ G2 (*P* = 0.149). Patients who received combined immunotherapy had significantly higher incidence of any irAEs (*P* = 0.020) and ≥ G2 irAEs (*P* = 0.022) than patients receiving single-agent immunotherapy. Furthermore, there were no significant differences in the occurrence of both any irAEs (*P* = 1.000) and irAEs ≥ G2 (*P* = 0.603) between nivolumab and pembrolizumab.

## Discussion

To date, ICIs have been developed for clinical treatment in a variety of malignancies including NSCLC. A great deal of effort has been devoted to find predictive biomarkers to identify the patients who will have the best response to ICIs. As shown in previous studies, PD-L1 expression, tumor lymphocytic infiltration, TMB and microsatellite instability might be closely related to outcomes of immunotherapy 17-20. However, they are not the perfect predictors. The expression of PD-L1 is determined by immunohistochemistry (IHC), yet IHC can be performed in different technical staining platforms using different antibodies, which leads to different definitions of PD-L1 positivity. In addition, the heterogeneity and dynamics of PD-1/PD-L1 expression in tumors should also be taken into consideration. Furthermore, it is difficult to determine the lymphocytic infiltration and the expression of PD-L1 by core biopsy samples. Thus, more sensitive and specific clinical biomarkers are urgently needed to predict the prognosis of patients undergo ICIs therapy. Given that patient clinical features and blood samples are the accessible and intuitive clinical indicators, we explored the impact of clinicopathological factors and blood examination results on the prognosis of immunotherapy in advanced NSCLC.

Several studies have investigated biomarkers that can predict the response to ICIs. To date, no clear evidence has shown that ICI treatment response is affected by the patient age, sex and ethnicity 21. It has been reported that smoking history may correlate with a higher response rate to ICIs treatment by increasing the mutation load of the tumor 22, 23. In addition, studies have shown that histologic markers of NSCLC may also be associated with therapeutic response of anti-PD-1 22-24. Furthermore, Cortellini et al. suggested that patients with poor ECOG score (≥ 2) have a lower incidence of irAEs of any grade, which may lead to an adverse prognosis 25. As reported previously, NSCLC with single organs metastasis exhibited generally survival benefit compared with the tumor with multiple organs metastasis, that attributed to lower tumor burden and local intervention to patients with a limited number of metastases 26. Increasing evidence has also shown that multiple organs metastasis might be a negative factor for outcome of anticancer therapy. A latest meta-analysis indicated that the NSCLC patients with multiple organs metastasis had higher risk of hyperprogressive disease (HPD) after treatment with ICIs, that significantly correlated with worse OS 27. In this present study, we also provided the evidence that the number of metastatic organ sites was associated with the outcomes of ICIs, that we should consider in clinical practice.

As a less invasive examination, reports of blood-based biomarkers to predict ICIs treatment of advanced NSCLC have so far been rare. Bagley et al. suggested that the pre-treatment NLR might predict the response of patients with NSCLC treated with nivolumab 24. Kasahara et al. reported that the Glasgow prognostic score (GPS) calculated using C-reactive protein and albumin concentrations after 1 month of anti-PD-1 treatment was closely related to clinical response, while pre-treatment GPS was not 25. In addition, they also found that post-treatment NLR, but not pre-treatment NLR, was associated with the efficacy of anti-PD-1 15. Consistent with this result, no correlation between pre-treatment NLR values and PFS in NSCLC patients with ICIs treatment was found in our study, and we observed that the high NLR of the fourth cycle of treatment was closely related to inferior PFS. As reported in previous studies, the elevated LDH may predict poor prognosis in NSCLC patients received anti-PD-1 treatment 28, 29. However, we did not find the correlation between LDH and patients PFS in present study. In addition, we also found that low levels of CEA before treatment were related to prolonged PFS. The reasons for the difference in results we considered as follows: 1) the cutoff values of LDH (245 IU/L; 240 IU/L) used in studies were different 28, 29; 2) the number of patients in our study was relatively small.

The irAEs brought by widely use of ICIs have become an increasingly concerned issue in clinical practice. Previous studies reported the irAEs including skin, endocrine, gastrointestinal, hepatobiliary system and others. In our study, we newly found that patients receiving ICIs may also have hemoptysis and cardiac system disfunction. Regarding the data in our study, irAEs (≥ G2 / < G2) were confirmed to be the independent predictors of superior PFS, suggesting that patients who experienced ≥ G2 irAEs had a better treatment response. In line with this, previous studies suggested a similar association in patients with advanced melanoma and NSCLC 11, 12, 30-33. Taken together, although irAEs exist as therapeutic adverse effects in patients receiving ICIs and requires special attention from physicians, it may indicate that the patient's immune function is intact and has a better response to immunotherapy. Therefore, balancing the advantages and disadvantages of irAEs will greatly improve the therapeutic effect of ICIs therapy.

There were several limitations in our study. First, our study was retrospective and single-institutional with a limited number of patients. Second, treatment time as well as treatment options are chosen by the attending physician, thus, there is no standardization between patients. Third, the PD-L1 expression was positive in 16 patients (26.2%), negative in 2 patients (3.3%) and not available in 43 (70.5%). Nevertheless, emerging evidence suggests that PD-L1 expression does not fully represent whether patients are effective for anti-PD-1/ anti-PD-L1 treatment. In contrast, some patients with low or no PD-L1 expression benefited significantly from anti-PD-1 / anti-PD-L1 therapy 9, 10.

In conclusion, patients with metastatic sites ≥ 3 and higher CEA levels may not benefit from ICIs therapy. The occurrence of irAEs (≥ G2), best response and age < 65 yr were independent predictors of ICIs efficacy in advanced NSCLC. Early identification of patients who respond to ICIs treatment, not only reduce the cost of treatment and the risk of severe irAEs, but also help to greatly improve the effectiveness of ICIs.

## Figures and Tables

**Table 1:** Clinicopathological characteristics of patients

Characteristic	n (%)
	61
**Age (y)**	
≥ 65 yr	11 (18)
< 65 yr	50 (82)
**Sex**	
Male	38 (62.3)
Female	23 (37.7)
**Smoking history**	27 (44.3)
**Histology**	
Squamous cell carcinoma	16 (26.2)
Adenocarcinoma	45 (73.8)
**Clinical stage**	
III B	6 (9.8)
IV	55 (90.2)
**PS**	
0-1 2	60 (98.4) 1 (1.6)
**No. of metastasis sites**	
≥ 3	10 (16.4)
< 3	51 (83.6)
PFS, month*	5.2 (1.7-7.4)
**Response**	
PR	18 (29.5)
SD	24 (39.3)
PD	13 (21.3)
NE	6 (9.8)
**Disease control rate, %**	68.9
**Line of immunotherapy**	
First	17 (27.9)
Non-first	44 (72.1)
**Type of immunotherapy**	
Nivolumab	24 (39.3)
Pembrolizumab	6 (9.8)
Atezolizumab	27 (44.3)
Nivolumab+Ipilimumab	4 (6.6)
**irAE**	
Any irAE	24 (39.3)
I-II grade	20 (83.3)
III-IV grade	4 (16.7)

Abbreviations: PS, Performance Status; PFS, Progression Free Survival; *Data shown as (25 percentile to 75 percentile); PR, partial response; SD, stable disease; PD, progression disease; NE, not evaluated; irAE, immune-related adverse event.

**Table 2:** Univariate Analysis of clinical characteristics and blood index on PFS and OS

Characteristic	PFS			OS		
	*P*-value	HR	95% CI	*P*-value	HR	95% CI
**Clinic index**						
Sex (male/female)	0.081	1.647	0.941-2.883	0.737	1.124	0.567-2.229
Age (<65 / ≥ 65 yr)	0.320	1.425	0.708-2.867	**0.001**	3.878	1.686-8.921
Metastasis (< 3 / ≥ 3)	**0.039**	2.146	1.038-4.436	**0.012**	2.826	1.256-6.361
Smoke (never / ever)	0.399	0.786	0.449-1.376	0.852	0.937	0.472-1.858
Histology (SC / AD)	0.794	1.086	0.585-2.017	0.350	1.489	0.646-3.435
Clinical Stage (IIIB / IV)	0.805	1.123	0.446-2.832	0.839	1.115	0.389-3.198
PS (0-1/ ≥ 2)	0.636	0.883	0.528-1.477	0.419	1.320	0.673-2.590
Therapy strategy^★^	0.645	0.786	0.282-2.189	0.370	0.516	0.122-2.189
Line of therapy (1/2/3/5)	0.128	1.319	0.923-1.884	**0.016**	1.699	1.105-2.613
Type of immunotherapy^※^	0.582	0.886	0.575-1.364	0.611	1.098	0.767-1.571
irAE (class I) *	0.060	0.573	0.321-1.023	0.085	0.512	0.239-1.098
irAE (class II) **	**0.016**	0.395	0.185-0.843	**0.025**	0.196	0.047-0.818
Response #	**<0.001**	9.203	4.738-17.877	**0.009**	1.978	1.181-3.310
**Blood index**						
NLR C1 (< 5 / ≥ 5)	0.431	0.709	0.301-1.670	0.520	0.676	0.205-2.226
NLR C4 (< 5 / ≥ 5)	**0.002**	3.060	1.521-6.150	0.194	1.741	0.754-4.022
PLT (≤ 350 / > 350×109/L)	0.532	0.794	0.386-1.635	0.755	0.868	0.357-2.110
LDH (≤ 369 / > 369 U/L)	0.965	0.977	0.349-2.734	0.163	2.118	0.737-6.087
CEA (≤ 5 / > 5 ng/ml)	**0.014**	0.437	0.225-0.846	0.086	0.513	0.240-1.099

HR: Hazard ratio; SC: Squamous cell carcinoma; AD: Adenocarcinoma; Therapy strategy^★^: mono-immunotherapy or doublet-immunotherapy; Type of immunotherapy**^※^**: anti-PD-1/anti-PD-L1/anti-PD-1+anti-CTLA4; *: no irAE/any irAE; **: irAE<G2 /irAE≥ G2; Response **^#^**: PR/SD/PD; NLR: neutrophil lymphocyte rate; PLT: Platelet; LDH: Lactate dehydrogenase; CEA: Carcinoembryonic antigen.

**Table 3:** Multivariate Analysis of clinical characteristics and blood index on PFS and OS

Characteristic	PFS			OS		
	*P*-value	HR	95% CI	*P*-value	HR	95% CI
**Response to therapy**	**<0.001**	10.435	3.677-29.61	0.143	2.180	0.769-6.179
**irAE (≥2/<2)**	**0.009**	0.240	0.082-0.704	0.081	0.237	0.063-1.176
**Metastasis**	0.455	2.480	0.228-26.91	0.633	0.724	0.192-2.728
**Age**				**0.001**	5.450	1.982-14.98
**Line of therapy**				0.980	0.979	0.187-5.122
**CEA**	0.169	0.567	0.253-1.273			
**NLR C4**	0.324	1.630	0.617-4.305			

**Table 4:** Clinical and treatment characteristics associated with the response to ICIs

Characteristics	Treatment Response			* P*-value
	PR (n=18, %)	SD+PD (n=37, %)		
**Age (y)**				0.702
≥65 yr	2 (11.1)	7 (18.9)		
<65 yr	16 (88.9)	30 (81.1)		
**Sex**				0.462
Male	13 (72.2)	23 (62.2)		
Female	5 (27.8)	14 (37.8)		
**Smoking history**				0.769
Non-smoker	10 (55.6)	19 (51.4)		
Smoker	8 (44.4)	18 (48.6)		
**Histology**				0.08
Squamous cell carcinoma	8 (44.4)	8 (21.6)		
Adenocarcinoma	10 (55.6)	29 (78.4)		
**Clinical stage**				1.00
IIIB	2 (11.1)	4 (10.8)		
IV	16 (88.9)	33 (89.2)		
**No. of metastasis sites**				1.00
≥3	2 (11.1)	4 (10.8)		
<3	16 (88.9)	33 (89.2)		
**Treatment strategy**				**0.035**
Mono-immunotherapy	14 (77.8)	36 (97.3)		
Immuno+chemotherapy	4 (22.2)	1 (2.7)		
**irAE (class I)**				0.391
Any irAE	9 (50)	14 (37.8)		
No irAE	9 (50)	23 (62.2)		
**irAE (class II)**				0.614
≥2	5 (27.8)	8 (21.6)		
<2	13 (72.2)	29 (78.4)		
**Progression**				**0.01**
No	7 (38.9)	3 (8.1)		
Yes	11 (61.1)	34 (91.9)		
**CEA**				0.44
> 5	4 (22.2)	14 (37.8)		
≤ 5/NA	14 (77.8)	23 (62.2)		
**NLR C4**				0.296
≥ 5	2 (11.1)	10 (27)		
< 5/NA	15 (88.9)	27 (73)		
**PLT**				0.713
> 350×10^9^/L	10 (55.6)	19 (51.4)		
≤ 350×10^9^/L	8 (44.4)	18 (48.6)		
**LDH**				0.59
≥ 369 IU/L	2 (11.1)	24 (64.9)		
< 369 IU/L	16 (88.9)	13 (35.1)		

**Table 5:** Profiles of Immune-related Adverse Events

Category	Number of patients (%)			
	Total (%)	anti-PD-1	anti-PD-L1	anti-PD-1+anti-CTLA4
	N=61	N=30	N=27	N=4
**Any**	24 (39.3)	15 (50)	5 (18.5)	4 (100)
Grade 1	9 (14.7)	6 (20)	2 (7.4)	1 (25)
Grade 2	11 (18.0)	8 (26.7)	2 (7.4)	1 (25)
Grade 3	4 (6.6)	1 (3.33)	1 (3.7)	2 (50)
Grade 4	0	0	0	0
**Skin**				
Rash	5 (8.2)	3 (10)		2 (50)
Skin desquamation	1 (1.6)	1 (3.3)		
**Endocrine**				
Hyper/hypothyroidism	4 (6.6)	3 (10)		1 (25)
Respiratory system				
Immuno-related pneumonia	5 (8.2)	5 (16.7)		
Haemoptysis	3 (4.9)	2 (6.7)	1 (3.7)	
**Hepatobiliary system**				
ALT/AST elevation	5 (8.2)	2 (6.7)	1 (3.7)	2 (50)
**Gastrointestinal**				
Amylase increase	1 (1.6)			1 (25)
Nausea/vomiting	1 (1.6)		1 (3.7)	
Diarrhea	1 (1.6)			1 (25)
Cardiac dysfunction				
Premature ventricular beats	1 (1.6)			1 (25)
Myocardial ischemia	1 (1.6)	1 (3.3)		
**Other**				
Fever and Fatigue	7 (11.5)	2 (6.7)	4 (14.8)	1 (25)
Anemia	2 (3.3)	1 (3.3)		
Thrombocytopenia				1 (25)
Albumin reduction		1 (3.3)		
Dizziness	1 (1.6)		1 (3.7)	
Hyponatremia	2 (3.3)	1 (3.3)		
Hypochloremia	1 (1.6)	1 (3.3)		
Creatinine increase	1 (1.6)	1 (3.3)		
Uric acid increase	1 (1.6)		1 (3.7)	
Xerophthalmia	1 (1.6)	1 (3.3)		
Pigmentation	1 (1.6)	1 (3.3)		
